# Detection of *Coccidioides posadasii* in a patient with meningitis using metagenomic next-generation sequencing: a case report

**DOI:** 10.1186/s12879-021-06661-z

**Published:** 2021-09-17

**Authors:** Yuqiao Mao, Xia Li, Haibo Lou, Xiaoyu Shang, Yanjun Mai, Lan Yang, Fuhua Peng, Xihua Fu

**Affiliations:** 1 Department of Infectious Diseases, Guangzhou Panyu Central Hospital, Guangzhou, China; 2 Department of Bioinformatics, Hugobiotech Co., Ltd., Beijing, China; 3 Department of Neurology, Third Affiliated Hospital of Sun Yat-Sen University, Guangzhou, China

**Keywords:** Coccidioidomycosis, Meningitis, Metagenomic next-generation sequencing (mNGS)

## Abstract

**Background:**

Coccidioidomycosis is a systemic infection caused by dimorphic fungi *Coccidioides* spp. endemic to Southwestern United States and Central and South America. A history of residence and travel in these areas is essential for the diagnostic of coccidioidomycosis, which has highly variable symptoms ranging from asymptomatic to severe, disseminated infection, and even death. Immunocompromised patients of coccidioidomycosis experience a high risk of dissemination, chronic infection, and mortality. Meningitis is one of the most deleterious coccidioidomycosis and can cause various life-threatening complications.

**Case presentation:**

Here we report a case of *Coccidioides posadasii* meningitis in a 49-year-old female who returned to China after one and a half years residence in Los Angeles, USA. The repeated routine cultures using CSF for bacteria or fungi were all negative. To hunt for an infectious etiology, the state-of-the-art technology metagenomic next-generation sequencing (mNGS) was then utilized, suggesting *Coccidioides posadasii*. Organizational pathological examination and polymerase-chain-reaction (PCR) results subsequently confirmed the mNGS detection.

**Conclusion:**

To our knowledge, cases for coccidioidal meningitis have been rarely reported in China. While global travelling may spread this disease across continents and make the diagnosis more difficult. mNGS can detect almost all known pathogens with high sensitivity and specificity, especially for uncommon pathogen, such as *Coccidioides posadasii* in China.

## Background

Coccidioidomycosis is a mycosis commonly caused by *Coccidioides immitis* and *Coccidioides posadasii*, which only spreads in the Southwestern United States and Central and South America [1–3]. Primary host exposure to *Coccidioides* is mainly via inhalation of aerosolized arthroconidia upon soil disruption [4]. Coccidioidomycosis commonly manifests as an asymptomatic infection or a mild respiratory infection in humans and other vertebrate hosts [5]. However, the disease can also cause disseminated infection, progressive pulmonary infection, and infections in other organs including skin, bone, brain, and meninges [6]. In addition, coccidioidomycosis presents diverse symptoms among different patients and along different disease stages. Both clinical and imageological presentations of coccidioidomycosis are not typical, making it easy to be misdiagnosed [6]. This disease is rarely reported in China. There are 39 reported cases of coccidioidomycosis in China during 1958–2017 [7]. The number of coccidioidal meningitis cases are even smaller.

Here we report a case of *C. posadasii* infected meningitis in a 49-year-old female who returned to China after one and a half years residence in Los Angeles, USA. During this period, the patient had tidied up the flowers and other plants in the garden for several times, probably inhaled *Coccidioides* spores, and then got infected. Traditional clinical diagnostic assays failed to identify the pathogen. Metagenomic next-generation sequencing (mNGS) was then introduced. mNGS is a new approach that can rapidly identify almost all known pathogens in clinical samples with high sensitivity and specificity. It has been used in many clinical practices, including meningitis [8]. In this study, *C. posadasii* was finally detected by mNGS from cerebrospinal fluid (CSF) sample.

## Case presentation

A 49-year-old female patient complained of fever and headache for 32 day was hospitalized in the Department of Infectious Diseases of Guangzhou Panyu Central Hospital, Guangdong, China, on October 29th, 2020. She had lived for one and a half years in Los Angeles, USA and just returned to China 2 days before. While living in the United States, she traveled around Los Angeles and trimmed plants for several times in the garden.

The symptoms included a sudden onset of fever (39 °C), severe headache, and rash on her face and back while she was in Los Angeles on September 27th. The patient went to local hospitals several times, but computed tomography (CT) showed no abnormities on her head. She received symptomatic treatments, but had not fully recovered.

When admitted to our hospital, the patient was with headaches, fevers, vomiting, and maculopapular rashes (1.5 cm × 1.5 cm) on the face and back. Her white blood cell count (WBC), erythrocyte sedimentation rate (ESR), and C-reactive protein (CRP) were slightly higher than the normal range, but her electrolytes was slightly lower. CT examinations revealed small nodules in the posterior apex of her left upper lobe and lateral segment of middle lobe of right lung, but no abnormalities in head.

**Fig. 1:**
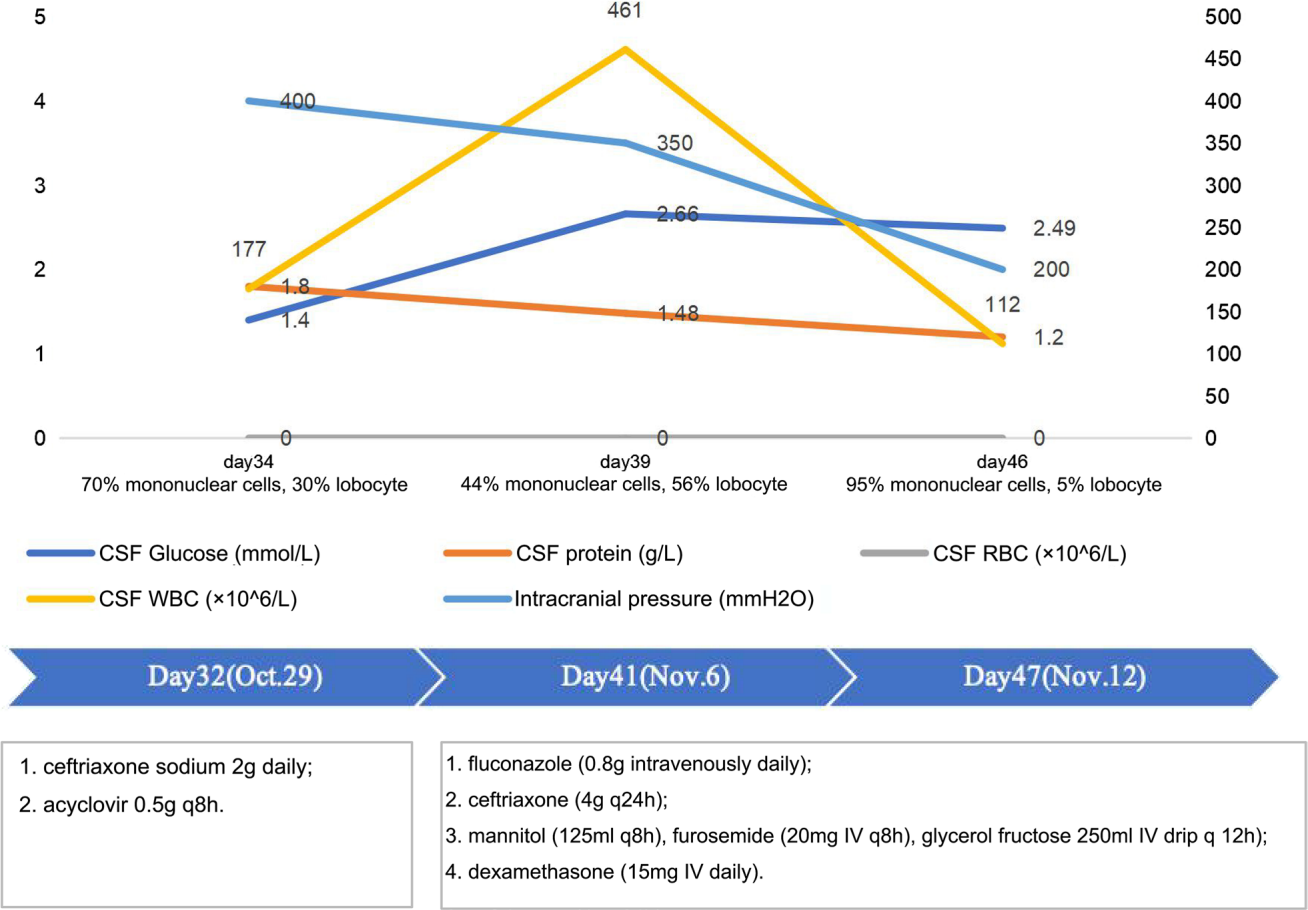
CSF investigation and antimicrobial drugs. After the adjustment of the treatment, the CSF factors including glucose content, protein content, WBC, and opening pressure gradually returned to normal

The empiric combinatory therapy (ceftriaxone sodium 2 g daily and acyclovir 0.5 g q8h) for both viral and bacterial meningitis was given. A lumbar puncture was performed on day 34. The CSF opening pressure was greater than 400 mmH_2_O. CSF analysis demonstrated positive pandy test and negative cryptococcal antigen, gene Xpert MTB/RIF assay, and culture result (Fig. 1). Drug dehydration and other symptomatic treatments were added. But there was no relief of the symptoms.

**Fig. 2:**
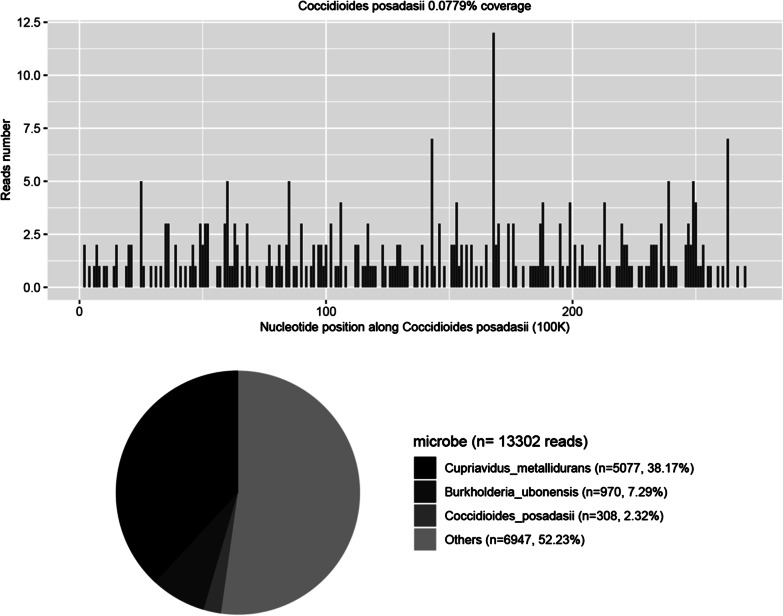
The mNGS result of *C. posadasii*. 2.32% of bacterial reads corresponded to *C. posadasii* with a coverage of 0.0779%

**Fig. 3:**
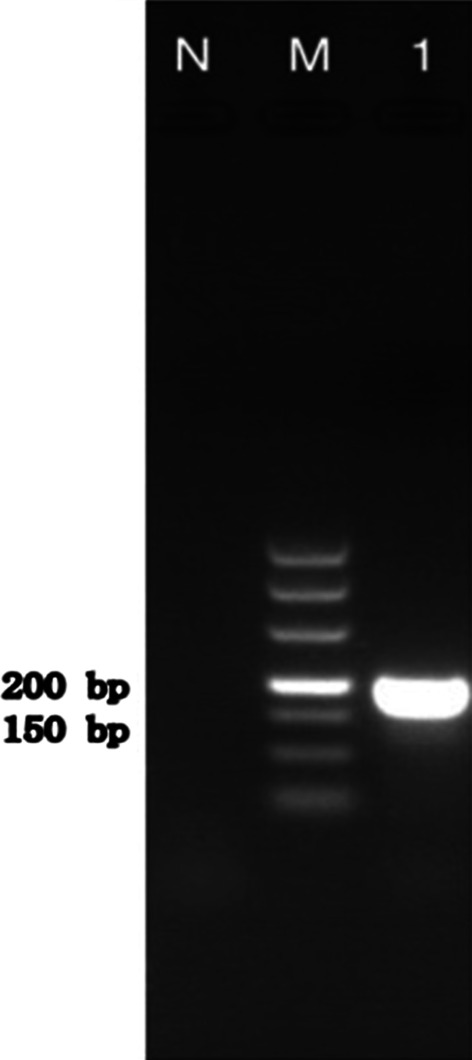
PCR detection of *C. posadasii*. Lane 1: 195-bp polymerase chain reaction product of *C. posadasii*; Lane M: DNA ladder; Lane N: Negative control

**Fig. 4:**
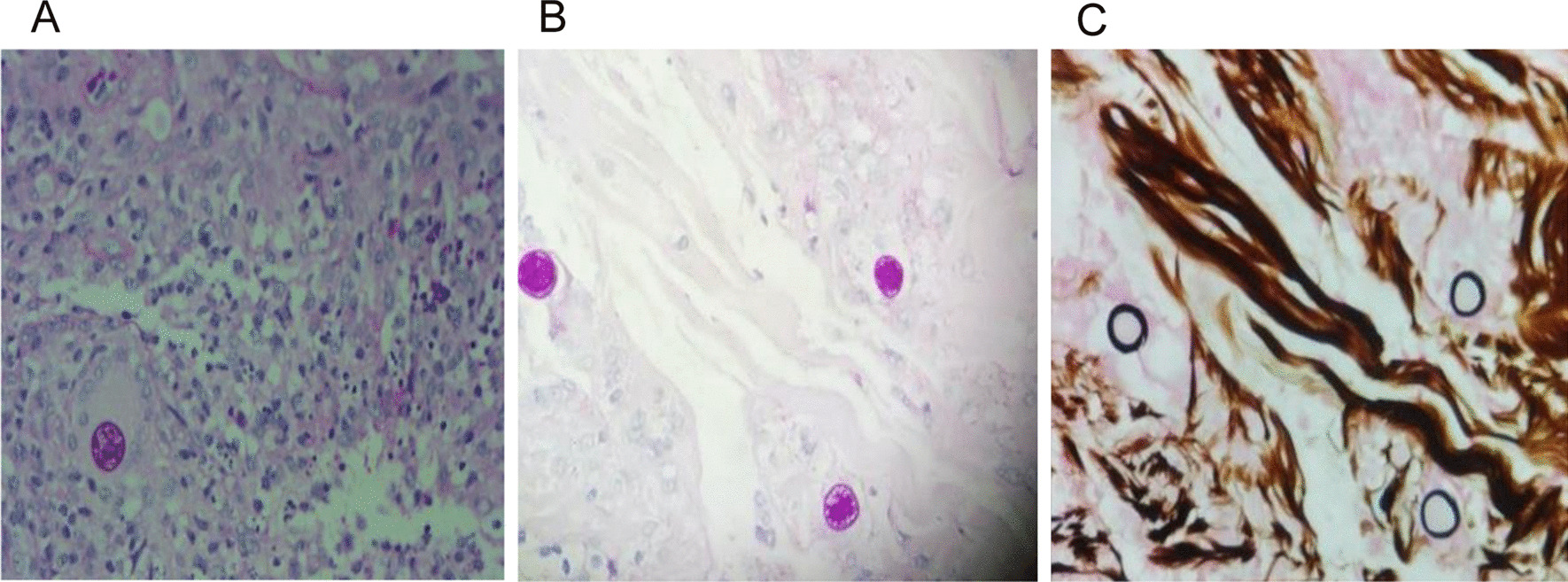
Biopsy of back rash subcutaneous tissue. **A** HE stain, and **B** PSA stain, coarse spheroids were covered by double-layer envelope, with endospores inside, and surrounded by neutrophils and eosinophils; **C** Hexamine silver stain, positive spheroids can be seen in the pathology

A lumbar puncture was repeated on the 39th day (Fig. 1). Autoimmune encephalitis spectrum results were positive of the anti-MOG-IgG antibodies for the serum and the CSF (1:32 and 1:3.2, respectively). To further identify the pathogen, PACEseq mNGS (Hugobiotech, Beijing, China) of CSF and blood was performed on a Nextseq 550 platform (Illumina, California, USA). A total of 308 unique sequence reads were finally aligned to the *C. posadasii* genome in CSF, which showed a dominant abundance of 2.32% in all microbial species after excluding the human reads from the total gene pool (Fig. 2). The blood mNGS showed negative of the pathogen. A targeted PCR of *C. posadasii* and Sanger sequencing were subsequently applied, which finally confirmed the mNGS detection of CSF (Fig. 3). In addition, the tissue biopsy of the patient’s back rash indicated HE Stain, PAS stain (+), and hexamine silver stain (+) (Fig. 4), which were also in line with coccidioidomycosis. Considering the abnormal signals in the bilateral parieto-occipital lobes and the meninges by magnetic resonance imaging (MRI) and magnetic resonance angiography (MRA), the patient was finally diagnosed with *C. posadasii* infected meningitis.

The treatment was adjusted with fluconazole (800 mg intravenously daily) and ceftriaxone (4 g q24h). Other symptomatic treatments were also performed, such as mannitol (125 ml intravenously q8h) to reduce intracranial hypertension. The detailed treatments were shown in Fig. 1.

The patient gradually recovered over the next 7 days. The body temperature returned to normal, and the headache and dizziness relieved. Repeated lumbar puncture revealed the pressure at 200 mmH_2_O (Fig. 1). The patient's vital signs were stable. The patient was then transmitted to Third Affiliated Hospital of Sun Yat-Sen University for antifungal treatment with altericin B combined fluconazole. One month later, the patient’s symptoms improved and was finally discharged. She was required oral fluconazole. On February 2021, reexamine of the patient found high blood fat with intrajugular venous thrombosis and head sinus. The patient was given levarabban, oral anticoagulant therapy, fluvastatin, and oral calcium supplements. After 3-month follow-up, her headache aggravated with a increase intracranial pressure. The patient then received dehydration treatments.

## Discussion and conclusions

Coccidioidomycosis is a systemic fungal infection caused by *C. immitis* or *C. posadasii*, which usually exist in hot and dry environment. In endemic areas, farmers and construction workers who frequently touch the soil have a higher prevalence [9]. Cases with disseminated coccidioidomycosis had a high risk (~ 50%) to develop into coccidioidal meningitis within weeks to months [10]. As one of the most deleterious coccidioidomycosis, coccidioidal meningitis can result in various life-threatening complications, including vasculitis, cerebral infarction, hydrocephalus, and Spinal arachnoiditis [11, 12]. Coccidioidal meningitis can also cause symptoms such as chronic persistent headaches, fever, nausea, and vomiting, and it is uniformly fatal in patients without treatment [6, 13]. In this study, the patient had lived in the United States for a long time with a history of touching soil and organizing flowers and plants in the garden. She experienced repeated fever, headache, and rash after the onset. Analysis of CSF and imaging examinations revealed infectious lesions. However, routine clinical tests, including culture did not find the pathogen. Finally, mNGS was used and successfully revealed coccidioidomycosis, which was later confirmed by PCR and biopsy results.

The routine diagnosis of coccidioidomycosis include immunologic assays, culture, and histopathology of tissues. However, there are many limitations. For example, serology is most commonly used, but the sensitivity is not ideal in the early diagnosis, especially for immunocompromised patients. mNGS is a new molecular diagnostic method which performs many advantages comparing with traditional methods. It can rapidly detect almost all known pathognes in one run with high sensitivity and specificity [14–17]. mNGS has been applied in pathogens detection in various clinical practices, such as meningitis, sepsis, and acute respiratory infection, especially for diseases infected by new, rare, difficult-to-culture, and mixed pathogens [6, 8, 18]. Due to the really low incidence of coccidioidomycosis in China, it was not first considered. So, the traditional methods failed to diagnose the pathogen. Fortunately, mNGS was used and finally detected the pathogen, which helped the diagnosis and timely treatment.

Due to the high mortality (90% in 1 year and 100% in 2 years) of coccidioidal meningitis in untreated patients, the early identification of pathogens and the timely antibiotic treatment is the key for a favorable prognosis [19]. However, the early diagnosis of coccidioidal meningitis remains a clinical challenge. This case indicated the possibility of mNGS as an early diagnostic measure for coccidioidomycosis, and proposes to employ this procedure in patients with relative symptoms and travel history.

## Data Availability

The data of this manuscript is available at http://ngdc.cncb.ac.cn/omix/preview/AAFXROl8, Reference Number PRJCA006430.

## Abbreviations

WBC: White blood cell count
CRP: C-reactive protein
ESR: Erythrocyte sedimentation rate
CSF: Cerebrospinal fluid
CT: Computed tomography
MRI: Magnetic resonance imaging
MRA: Magnetic resonance angiography
PCR: Polymerase chain reaction
PPI: Proton-pump inhibitor
